# Crossing boundaries in the delivery of healthcare – a qualitative study of an eHealth intervention in relation to boundary object theory

**DOI:** 10.1177/20552076231196970

**Published:** 2023-08-30

**Authors:** Trust Saidi, Erlend Mork, Sofie Aminoff, Fiona Lobban, Kristin Lie Romm

**Affiliations:** 1 Centre for Connected Care (C3), 155272 Oslo University Hospital, Oslo, Norway; 2 Centre for Technology, Innovation and Culture, 6305 University of Oslo, Oslo, Norway; 3 Athena Institute, 1190 Vrije University Amsterdam, Amsterdam, the Netherlands; 4 Early Intervention in Psychosis Advisory Unit for South-East Norway, Division of Mental Health and Addiction, 155272 Oslo University Hospital, Oslo, Norway; 5 NORMENT, Division of Mental Health and Addiction, Oslo University Hospital & Institute of Clinical Medicine, 6305 University of Oslo, Oslo, Norway; 6 Spectrum Centre for Mental Health Research, Division of Health Research, Faculty of Health and Medicine, 4396 Lancaster University, Lancaster, UK

**Keywords:** Boundary object, REACT-NOR, supporters, caregivers, psychosis

## Abstract

With the increasing trend of digitalisation in the health sector, eHealth is being deployed to facilitate interaction between health professionals and service users without physical contact or close proximity. It became prominent during the COVID-19 era when mobility for physical meetings was restricted. Focusing on a video-supported digital toolkit, REACT-NOR, this study explored the experiences of caregivers and supporters in relation to the notion of boundary object. In-depth semi-structured interviews were conducted with 10 supporters and 11 caregivers to gather first-hand experience on the use of the digital tool. It emerged from the study that the use of REACT-NOR made a huge difference for the involved parties by bridging the knowledge gap between supporters and caregivers. The use of the video in particular was useful in engaging and emotionally connecting the supporters and caregivers, resulting in an exciting digital experience. The effectiveness of the digital tool can be explained in the context of a boundary object in that it facilitated the processes of transferring, translating and transforming knowledge. The tool exhibited the attributes of dynamism, flexibility, standardisation and shared structure, which resonates with the notion of a boundary object. An understanding of how boundary objects work is crucial especially with remote care, as depicted in this study, due to the fact that the transfer of knowledge involves multiple processes such as sharing of new and existing knowledge, translation to make it accessible to others and transformation to render it usable across different boundaries.

## Introduction

Globally, eHealth is emerging as an essential component of healthcare delivery that is used to complement in-person support. World Health Organization^1^ defines eHealth as the cost-effective and secure use of information and communications technologies in support of health and health-related fields, including healthcare services, health education and knowledge. eHealth may facilitate interaction among health professionals and service users without physical contact or close proximity^2,3^ and optimise the use of resources, improve user experience and represent the future of medical practice by expanding care to those with limited access to healthcare services.^4^ This is important as healthcare is highly specialised and knowledge-intensive, with organisational, professional and disciplinary boundaries.^5^ It renders collaboration and coordination across these boundaries essential. The high levels of differentiation between professionals and organisational units call for new forms of organising work across disciplines and medical specialities, which in turn changes the dynamics of social practices.^6,7^

Boundaries in healthcare came under the spotlight by the outbreak of COVID-19 as healthcare across the world grappled with the daunting challenge of delivering services safely without overtly being exposed to the virus.^8–11^ It marked a gradual shift in the delivery of healthcare from the conventional model requiring physical presence of patients in health institutions, to a potentially more flexible and affordable one.^12,13^ This is in line with the United Nations’ sustainable development goals, especially goal 3: ensure healthy lives and promote well-being for all at all ages, and goal 10: reduce inequality within and among countries.^1^ However, coordinating and collaborating on the delivery of healthcare between service providers and recipients can be challenging considering the complexity of the systems which are composed of distinct boundaries, missions, institutions, professional roles, and modes of distributing resources.^14^ Digital platforms are increasingly being used as boundary-spanning objects to connect and support diverse and heterogeneous user groups such as clinicians, patients and caregivers.^15^

Several studies have been conducted on the notion of boundary objects (BOs) in healthcare. For example, an investigation was made on how the concept of BO can be used to bridge temporally sequential boundaries between different practice communities to facilitate the translation of research evidence into practice.^16^ Another study analysed how high-risk patient operated as a shared object of intervention in cross-sector collaboration using the boundary object theory.^14^ The previous studies have made significant contributions towards understanding the concept of BOs in the health sector, but there are still some research gaps, particularly on how the collaboration across disciplinary, professional, organisational and geographical boundaries plays out in a variety of settings and under different conditions.

Remarkably, there is a relatively small number of studies on the concept of BO in cross-boundary collaboration between healthcare research and practice.^17^ The extent to which the notion of BO goes from in-theory to in-use is contingent on contextual factors such as who was involved in the creation process and the level of co-production. It is argued that future research should focus on the interplay of boundary work beyond the confines of the clinical settings by incorporating spatial dimensions.^5^ Linked to this, a study indicated that there is a paucity of studies on how the concept of BO could be deployed to facilitate the translation of research evidence between non-intersecting communities.^16^ Relatives to people with serious mental illness, are a group that often feels overlooked, left out and even stigmatised,^18^ as they too seldom are involved in the clinical work. Yet, they have responsibilities, and as such, are part of a community that have overlapping interests with the healthcare services. Previous research underlines the need for psychoeducation, problem-solving strategies, peer support and clinical guidance.^19^

Taking cues from previous studies, the current study attempts to expand the knowledge base by exploring the functionality of a web-supported e-learning program used in combination with video consultation by focusing on the collaboration between clinicians and caregivers as non-intersecting communities situated beyond a hospital setting. Specifically, our aims in this paper are two-fold (i) to explore how a supported web-based toolkit (REACT-NORV) and video consultation was experienced by caregivers and supporters, (ii) to assess the capabilities embedded in the toolkit in relation to BO theory and discuss implications related to future eHealth development.

## Theoretical framework

### Boundary objects

The notion of BOs as originally defined refers to ‘objects which are both plastic enough to adapt to local needs and the constraints of the several parties employing them, yet robust enough to maintain a common identity across sites’.^20^ For objects to become BOs, they must be used at the interface of different communities of practice to connect them.^21^ They bind the communities of practice together through a shared understanding of what they do, how they do it, and how they relate to other communities and their practices, resulting in the development of the same worldview or mental model.^22^ The notion of BOs exists in different forms. They can be physical objects, such as documents containing diagrams of the system architecture,^21^ or virtual objects which are stored in computers, databases, such as emails, websites, and electronic databases that can be transferred electronically.^23^ The plasticity of BOs is a crucial attribute as it facilitates communication between different communities of practice towards a common goal.^24,25^

BOs possess four characteristics: (a) BOs have a *shared structure* which enables them to serve as objects of action that actively link fields and stimulate dialogue.^24,26^ They inhabit a shared space, where the sense of here and there are confounded by connecting the boundaries to ensure that different (inter-)disciplinary perspectives overlap and interact.^26^ (b) BOs exhibit *interpretive flexibility* in that they have different meanings in different fields, but they are resilient enough to contribute to a mutual understanding of individuals.^20^ BOs provide multiple translation possibilities within cooperative arrangements.^27^ (c) BOs are *dynamic* in that they offer a range of uses from broad and unstructured to more tailored and precise.^26^ The dynamic structure of BOs render them usable strategically to disrupt, establish, or maintain institutional practices and hence they are an embodiment of institutional work.^28^ (d) BOs are characterised by *standardisation* of content, methods and measures that develop as they move between groups and across scales.^26^ This is achieved by maintaining a specific structure across groups, while not being limited by the ways information is interpreted and applied. The standardisation is anchored on agreed-upon rules and definitions that are designed to reduce inconsistencies and potentially conflicting practices.^29^ However, there are trade-offs in that when a BO is strongly structured, it can function as a coordinating mechanism in explicit knowledge communication yet it loses in creativity and tacit knowledge communication.^15^

The concept of BO is useful in transforming knowledge and changing practices across professional boundaries.^30^ This is achieved through three progressively complex processes namely *transfer, translation and transformation of knowledge*.^31^ Transfer of knowledge involves sharing of existing knowledge and making it accessible to others, translation refers to interpretation of that knowledge to make it accessible to others, while transformation means making knowledge usable across different boundaries.^32^ These functions of BOs, are made possible by acknowledging the existence of common knowledge that actors can use to share domain-specific knowledge.^31^ For example, through the translation role of BOs, the pragmatic boundaries between the partners are transformed and a shared understanding of partnering is developed resulting in communities of practice. This affirms the centrality of BOs, not only in understanding interactions across the limits of a particular setting, but also highlighting the necessity of seeing such objects as socially constructed, often idiosyncratic and always grounded in the local conditions governing interaction.^33^ In this study, we posit the notion of BO as an artefact to explain the interaction and translation that facilitates work and collaboration between heterogeneous groups.

#### BO in the delivery of healthcare

The concept of BO has been applied widely in healthcare to understand interprofessional collaboration and improve overall healthcare outcomes.^34^ By virtue of being highly specialised and knowledge-intensive organisations, healthcare organisations are characterised by organisational, professional, and disciplinary boundaries that mark the formal structure and division of work.^5^ In such settings, BO perform different roles namely facilitating collaboration, knowledge exchange, establishing shared meanings, and facilitating collective learning among clinicians, patients and caregivers.^35^ These roles are crucial in transcending knowledge boundaries of a syntactic, semantic, and pragmatic nature in the delivery of health, thereby contributing to the creation of common interests, meanings and lexicon.^16^ BOs can give rise to standardised practices where the principles embodied by a BO are embraced, mainstreamed and institutionalised and become the norm among the actors.^28,36^ This is important in healthcare as BOs are useful in mitigating language and knowledge boundaries between different disciplines.

In the health sector, BOs can facilitate constructive cooperation between sites or social systems. This organisational side of BOs allows stakeholders such as nurses, patients and caregivers to work together towards embedding and adoption of innovation in practice.^37^ The organisational focus of BO through their dynamic structure between ill-structured and tailored use can be important in bringing much-needed innovative transformation in healthcare.^37,38^ The transformation is dependent on how the three conceptually distinct, but interrelated forms of boundary work play out. The forms incorporate competitive boundary work which is anchored in mobilisation of boundaries to establish some kind of advantage over others, collaborative boundary work whose orientation is on the alignment of boundaries to facilitate collaboration and configurational boundary work in which patterns of differentiation and integration are manipulated with the aim of bringing some activities together, while others are kept apart.^6^ This renders BO to function as shortcuts to communication, as well as playgrounds for knowledge sharing among different communities of practice. The role of BOs is summed up as intermediaries in cross-collaboration, knowledge exchange as well as establishing shared meanings, and facilitating collective learning.^16^

With the growing call for integration of healthcare services, emphasis is shifting towards collaboration, communication and exchange of information across organisational boundaries to create a ‘seamless’ patient-centred healthcare system.^37^ To achieve this, the concept of BO is useful not only to understand how the healthcare system functions, but also to identify its problems and support collective learning.^16,35^ However, working with BO is not without challenges as the non-hierarchical collaborative form demands sufficient time and effort to build trust, contemplation, remembrance and other forms of emotional and embodied engagement.^39^ There are conditions that need to be met for BOs to travel among heterogeneous actors in healthcare. To ensure transferability, it is essential that local meanings are stripped off to give a universal outlook while abstract and standardised representation should be made contextually useable.^40^ In addition, the perceived possibilities for action that a BO offers to a user are crucial as they relate to the concept of affordability, which refers to the properties/qualities of an object or environment that suggest how it can be used or interacted with.^41^

Affordances are properties of the environment taken relative to an observer which is an embodiment of the inherent meaning embodied in objects and how it could affect behaviour.^42^ The concept revolves on the premise that perception is not solely based on the extraction of sensory information, but it also involves actionable possibilities or affordances.^43^ Affordance provides a theoretical position in understanding the mutual and functional interrelationship between the organism and its environment by emphasising an active engagement with the environment to extract meaningful information for guiding behaviour.^44^ In this study, the link between affordance and the concept of BO lies in their shared focus on interaction and interpretation. This emanates from the fact that affordances are about the inherent properties and potential actions that BOs offer, while the notion of BO is about the shared understanding and communication between different communities. Both concepts highlight the importance of context, perception and interpretation in design and interaction.

## Methods

### Setting and participants

This is a qualitative study based on data from the implementation process of REACT-NORV during the COVID-pandemic. The Norwegian healthcare system is public. Norway is divided into four health regions, and each region has several health trusts/hospitals. People with severe mental illness are prioritised and are accepted within a few days to the services. The trusts are asked to provide services for relatives, but they are not obliged to do so. They do have a duty to inform the relatives when the patient consent and give general advice. The original Relatives Education and Coping Toolkit (REACT) was developed as a peer-guided self-management intervention for caregivers of people with recent onset of psychosis at the Spectrum Centre for Mental Health Research in the United Kingdom, Lancaster University, and Lancashire Care National Health Services Foundation Trust.^45^ REACT-NOR is a Norwegian version previously pilot tested with a slightly different design, using telephone support by supporters trained in psychoeducational family therapy instead of peer support.^46^ Due to the COVID-19 pandemic, REACT-NOR was relaunched, this time with video support to enable safe support for caregivers, named REACT-NORV (REACT-NOR + Video). This enabled supporters and caregivers to meet and work with the toolkit while being able to see each other, and literally, be on the same (web) page. It turned out that the implementation of REACT-NORV has been a tremendous success during and after the pandemic. Currently, 13 of the 19 public health trusts offering psychiatric services across Norway, have educated 148 clinicians to support caregivers through REACT-NORV. This makes it an interesting object to explore which features that seem to appeal to both caregivers and supporters.

Due to the pandemic, clinicians trained in psychoeducational family therapy for psychosis were invited to be trained in using REACT-NORV as a tool to reach caregivers with information and provide them with tools to handle their situation. The ordinary psychoeducational family work was shut down in many places. Due to a national network of clinicians working with psychoeducational family therapy, we were able to reach several health trusts across Norway with information about the training. Trained supporters recruited caregivers in their own health trust. Some of the recruited participants lived in scarcely populated areas where long travel distances were an issue on beforehand.

When we refer to REACT-NORV in the following, we refer to both the web page, the video consultation and the support, as one service. All supporters (*n* = 22) and caregivers (*n* = 21) who had been recruited by 29 January 2021 were offered to participate. Ten supporters and 11 caregivers accepted the invitation. The participating supporters, seven women and three men, with a median age of 51 years (min 40–max 62) were mostly psychiatric nurses (8 of 10) and most (9 of 10) had more than 10 years of experience from mental health services. All were trained in psychoeducational family therapy. Half had supported one to two families in the REACT-NORV programme, the other half had supported between three and six families.

The participating carers, eight women and three men, with a median age of 55 (32–65) were all, except one, parents to a person with psychosis. Four of the 11 participants were currently living in the same household as their family member. The time since first treatment for psychoses varied greatly from two months to almost 16 years (median 1 year and 3 months). Most (seven) had received between eight and 11 sessions of support, two had less (four to seven sessions) and two more (11 to 15 sessions).

The study was presented to the Regional Committees for Medical Research Ethics (REC) South East Norway. The committee did not find the project to be medical or health professional research as understood by law (have to include patient outcome). They defined it as health service research, and hence it fell outside of the provisions of the Health Research Act (REC reference number 130328). The local data protection officer approved the project (Approval number 20/09173). All participants provided written informed consent in which they agreed to be interviewed and have the data analysed and published.

### Procedure

Receivers of REACT-NORV are caregivers to a person above 18 years of age, diagnosed with schizophrenia spectrum disorder by specialist mental healthcare services. They are either referred by the treating clinician, or self-referred. Each participant is assigned a supporter. After an initial face-to-face meeting, caregivers are offered regular digital sessions on a secure video line with their assigned supporters. The frequency of these meetings is mostly regulated by the caregivers, but during the current study period, it was regulated to a maximum of one session of 60 min per week for 16 weeks. The participants were introduced to the website reacttoolkit.no, which is open to everyone, during the first session.

### Intervention

The REACT-NORV toolkit consists of 13 modules: (a) What is REACT? (b) What is psychosis? (c) How to handle positive symptoms? (d) How to handle negative symptoms? (e) How to handle a crisis? (f) How to handle difficult behaviour; (g) coping with stress by thinking differently; (h) coping with stress by acting differently; (i) mental health services – how do I get the help I need? (j) Treatment options; (k) the future; (l) resources; and (m) terms and dictionary. The toolkit is rooted in psychoeducational family therapy. In addition, tasks connected to each theme are built in to help caregivers connect theory to their own circumstances. These tasks are based on cognitive behavioural therapy. The caregivers and the supporter share a screen and use the webpage actively during sessions. In between sessions, the caregivers are free to explore the web page and do tasks related to the themes they want to explore.

As previously described in the first pilot run in Norway,^47^ there were some modifications to the original UK program to reflect the Norwegian setting. The individuals in the case stories were given common Norwegian names, and the dictionary was translated and customised. The information about the mental healthcare system was altered to correspond with the Norwegian healthcare system. In the original UK version, caregivers were able to receive support and perform their tasks online through a chat function. We did not incorporate this feature due to a limited budget. Thus, REACT-NORV has never had any opportunity to draw on more advanced technological solutions, and the functionality is like a common web page.

### Supporters

The supporters are clinicians with varied professional backgrounds, but mostly psychiatric nurses or psychologists. Education and training in psychoeducational multi- and single-family groups (five days introduction class and monthly supervision for nine to 12 months) or cognitive behavioural therapy (two years programme), is a prerequisite to be able to join a REACT-NORV course. To be a REACT-NORV supporter, clinicians attend a digital course (3 × 3 h) and a 1.5 h biweekly digital supervision group for four months. The supporters are free to use their therapeutic knowledge actively during sessions. The training program builds on materials provided by the research team at Spectrum Centre for Mental Health Research. This was modified previously for the first Norwegian study using phone support. During the current video-based study, a web site about the project was developed to host material for both supporters and caregivers with videos/role plays and written information, as part of the implementation process.^48^

### Qualitative data

All participants in the current study were invited to a digital interview. They were interviewed individually by TS and KLR. TS and KLR were attending from different locations because of pandemic restrictions at the time of the interviews. TS is English speaking, so KLR assisted in translation if some of the participants encountered difficulties in understanding or speaking English. Our impression was that this was not a major problem, as most Norwegians speak and understand English well. All interviews were conducted during or within 12 weeks after the end of support. Each interview lasted for about 30 to 60 min. The interviews covered a broad range of questions ranging from the user engagement with the toolkit to the reflection on its effectiveness in addressing the challenges associated with providing support to patients suffering from psychosis. The interview guide for both the supporters and caregivers are presented in Appendix 1.

Researchers perspective: TS is a researcher interested in the relationship between health technologies and society. KLR is a psychiatrist. She is head of the Early Intervention in Psychosis Advisory Unit for South East Norway. She is a senior researcher on psychosis and service development, besides having extensive clinical experience. KLR has no training in psychoeducational family therapy.

### Analysis

The interviews were transcribed and coded systematically following several steps. TS, KLR, EM and SA read the interview transcripts separately to get an overview of the content. This was done to make the authors familiar with the data and allow for reflexivity, which according to Riach^49^ emphasises the importance of the knowledge construction in relation to the research objectives. The authors identified recurring themes from the interviews individually which were used as entry points in NVivo for sense making and making the data comprehensible. With the aid of NVivo software, seven themes were identified which were used for the first round of coding, similar to general open coding of qualitative data,^50^ whereby the empirical data is organised into discrete parts. The first author conducted the first round of coding using the themes and upon discussion with the fellow authors, it was noted that they were overlapping. This resulted in the themes being reduced to four. A second round of coding was done after the same four authors went through the thematic codes one by one. This was followed by axial coding^51^ where we drew connections between themes which we found central to the theory of BO. It involved using the codes to reveal themes embedded in the data to give directionality on the nexus between the toolkit and the functions of the BO.

## Results

From the coding process, four themes emerged and these form the structure in which the results of the study are presented.

### Opening a digital door between the services and caregivers, created an alliance

We found that the toolkit played a crucial role for both caregivers and supporters as it created a platform for interaction, as indicated by one of the caregivers in this quote:

It gives a good sense of togetherness. It gives good alliance that we can see each other on the screen and have a common goal to work with by following worksheets in the toolkit. (Caregiver #14)

Having supporters whom the caregivers of patients could link with for new knowledge and advice enhanced the connectivity. The toolkit served as a conduit that linked the caregivers to the supporters as indicated below. Being on screen did not seem to affect the possibility of building an alliance.

I find it surprisingly easier to establish a relationship with the caregivers of patients because I thought it would be a big obstacle to sit on the phone or screen talking, but as soon as I start helping them, describing their situation using the toolkit, connecting knowledge to their situation, pretty quickly, it seems like we are sitting in the same room, able to talk about things that I thought would be difficult digitally. (Clinician #1)

The two parties quickly formed a strategic alliance in that both the supporters and caregivers shared common goals which they intended to achieve by using the worksheets in the toolbox as depicted below:

It just shows that if you allow people to, you know, communicate on their terms, they are as engaged as everybody else, but you have to know how to reach people. So, this is just some new ways to reach people we might not have been able to reach previously. (Clinician #11)

It is explicit that the medium that is used to connect different actors is important as it can facilitate or hinder communication. The use of the video enhanced the functionality of the toolkit as indicated by several caregivers who argued that it helped them to maintain positive and productive communication. In an interview, one of the caregivers pointed out that:

Using video makes it easy to receive REACT. Otherwise, it would have been difficult to see how it could be done without video meeting because of long distances… (Caregiver #3)

However, connecting through the digital tool was not without challenges as firewalls in the hospital interfered with the process in some instances, resulting in erratic communication. Some supporters were forced to connect to the caregivers using phones and experienced that the situation limited the degree of connectiveness.

### Becoming educated by experts

The participants appreciated that the toolkit was compiled by experts in the field of psychosis, hence the content was developed from an informed perspective. The toolkit provided the caregivers of patients with relevant information that demystified psychosis by making it a condition that they could discuss without fear as indicated below:

The information I got through this program made me much safer. And it makes it easier to communicate about mental health or psychosis and to talk about symptoms. It is also possible for me to bring up the subject. (Caregiver #4).

The safety emanated from the fact that the toolkit was structured in such a way that it covered topical areas using concrete examples that resonated with the underlying needs of the caregivers of the patients. Thus, the caregivers found the toolkit to be useful because it addressed pertinent issues that they could easily relate to, thereby making it both educative and pragmatic:

There is so much information that I didn’t know so it gave me a lot of knowledge. So, when I got knowledge, I felt a bit safe to stay with my child because I am not a kind of a guy who likes to google things because you find everything, and you don’t know how to deal with it…. But in REACT I find a lot of information that was very useful for me. I learnt more, I know more about the situation and psychosis and how that is, and how the brain functions, what's happening and all the stigma, and all the things that you think about. (Caregiver #3)

Although mental health is stigmatised to such an extent that some family members may avoid talking about their experiences, the toolkit served as an intermediary that opened the seemingly sensitive subject for discussion. It allowed the caregivers to feel more confident in handling the situation. This was summed up by one of the supporters who argued that

What surprised me is that we established a relationship so fast, so it's easier to talk about more sensitive stuff for the families. It empowers them, they get new knowledge, and they get to use it and they get to test it out and they get to receive help and in how to use it to help the ones they care for and to help themselves. So, it does a lot more than I expected it to do (Clinician #5).

From the interview, it can be noted that the toolkit enabled the caregivers to access useful information from experts on how to handle their situation. With the aid from the supporters, the caregivers appropriated, the information to suit their situations which meant active engagement with the toolkit to derive more benefits from it.

### Empowerment by flexibility

As the caregivers of patients with psychosis need support in handling their situations, the supporters play an important role, but they do not impose how the caregivers should use the toolkit, as indicated below by a supporter:

The REACT toolkit is designed as a self-help tool to do home study, reading each chapter and worksheets that relate to the content of each chapter. We go through the worksheets; we go through what we have read, and we try to relate that to the situation they find themselves in. (Clinician #1)

The flexibility that comes with the use of the tool enables the caregivers to drive the process based on the goals that they want to achieve. This is important because the caregivers are empowered to explore how they can appropriate the tool to suit their peculiar situations. The supporters indicated that even during their interactions, the agenda for discussion is set by the caregivers of patients so that they take control of the whole process. This makes it possible to accommodate crisis management and switch to new topics when needed, as indicated below:

When we meet some are in a sort of a crisis or they are a bit confused or overwhelmed by the situation. The fact that the toolkit is split in chapters, it helps us to talk about one theme and it makes it easier, so it's the toolkit that helps with the structure, the worksheets, and the conversations. If we did not have the tool kit, we would focus on the here-now situation. With the kit, whatever would have happened from the last conversation would set the tone for what we would speak about, it makes it easier. (Clinician #2)

By virtue of having a structure in terms of the content, the toolkit proved to be comprehensive in that it covered a wide range of topics which the caregivers could choose from based on preference.

The toolkit makes sort of exploring together. And I am not the one to choose the topics, so that's good for the relationship. I think the caregivers experience more autonomy than if we were to make sessions at the clinic. So, I think that's good for relationships. (Clinician #9)

The way the toolkit is used differs from the traditional forms of interactions where experts who in this case are the supporters tend to determine what and how the recipients of information should be assisted. Instead, the toolkit allows for co-production of knowledge which empowers the caregivers of the patients to be responsible for making critical decisions on how they would want to use it.

### Convenience matters to all

For caregivers, REACT-NORV offers flexibility in terms of scheduling meetings while time is not wasted on travel. This was of special importance in rural areas, as revealed in the following interview:

For some people that have to travel, and they have to use time from work or from the patient or who else you know that it takes more time to go to the hospital because sometimes you have to drive for an hour or so. And it's you know, this is more flexible, they can sit at work, take an hour, and you know, just log on, and there we are, we start talking. So, it saves time as well. So, people are actually very positive to this way of doing things. (Clinician #11)

In several interviews, it emerged that travelling was demanding in terms of both time and financial resources especially if it involved a number of trips. Although having physical meetings was the preferred option by many supporters, the use of a digital platform was a flexible option, and it gave some positive side effects, as suggested by one of the supporters.

For me, I could have wished to see them in my office, to sit down and have coffee and have their toolkit to sit together. But for them, being at home was so relaxing. They could sit together, it was a parent, two parents and they were at home. And something come up in the week, as they have to work, and it was hard to get everything done. And could still meet me. And also, if the patient was in a bad mood, or they were worried about her, still they could meet me. (Clinician # 5)

However, both supporters and caregivers found it beneficial to have a first physical meeting to get to know each other. The use of the digital toolkit did not bring convenience to caregivers only, but also to the supporters who were very stretched by clinical work. Some of them were not able to find a slot for a meeting every fortnight because they lacked resources. In an interview, a supporter revealed that:

You are able to stay in touch with or in contact with families that you would have had to cancel appointment with, if you are meeting face to face. So, there are families that are maybe not highly prioritized enough to get the few hours you have available to meet face to face. (Caregiver #3)

## Discussion

The concept of BO has been applied in this study to explore the collaboration between caregivers and supporters across the boundaries of two worlds. Through the lens of a BO,^20^ the study reveals how the toolkit has been used in the dissemination of knowledge by providing a shared space and structure which served as a site of collaboration. However, we will argue that using video as a new addition to the toolkit made a huge difference for the involved parties as it provided affordances,^41^ which facilitated the caregivers to extract relevant information for action. By being able to see who they were talking to, share screen, and read text together, the boundary between supporters and caregivers was less pronounced. Several caregivers argued that the use of video enhanced their experience in connecting with the supporters. It enabled them to actively engage, and resulted in an enriched digital experience.

First, REACT-NOR provided a platform for bringing relatives and therapists together to link and interact in real-time. It served as a spanning object whereby the unmet needs of the relatives who were exposed to stressful situations were addressed with the support of the therapists. By connecting the relatives to patients, the toolkit facilitated the process of transforming knowledge and changing practices^41^ through what is described as three progressively complex processes namely knowledge exchange, establishing shared meanings, and facilitating collective learning. In this study, the connection was not limited to professional knowledge, but also tacit knowledge as the relatives drew from their lived experiences and became engaged as it was a person who was willing to receive it. They were able to connect and get to know each other through the video. This created an environment which made it easier for the relatives to open up and share information which could be deemed sensitive, especially using digital platforms. The toolkit thus typified a BO in that despite the geographic distance between the relatives and clinicians, it was able to facilitate the uptake, transfer and use of knowledge on psychosis, by working as a psychoeducational tool. Although some clinicians preferred physical meetings, they found the digital tool useful in building relationships in an environment characterised by time constraints. This can be explained in relation to the centrality of BO that they do not only promote interactions across the limits of a particular setting, but they also ground them in the local conditions governing the interaction.^33^ This could have been accomplished in a face-to-face setting too, by looking at the screen in the same room. The study design does not allow us to draw any conclusions about what works best. However, we can speculate the experience would be different as it would be easier to drift away from the toolkit when sitting in the same room. With a physical meeting, there is a likelihood that the stakeholders involved can feel more compelled to turn away from the screen and behave the way we normally do in a face-to-face meeting.

Second, the process of connecting the relatives to the therapists was done in a systematic way through the coordination of tasks which was made possible by the toolkit. The systematic approach was anchored on the existence of a shared structure and space,^24^ which is reminiscent of a BO. For example, the modular design of the toolkit allowed the relatives to navigate the topics of their interests and work towards a common goal by following the worksheets. It facilitated not only information sharing, emotional processing and problem solving, but also stress management which the relatives needed to cope with their situations. The design of the toolkit facilitated the process of bridging the gap between domain-specific knowledge to empower the relatives to handle the stressful situations. Although some relatives were overwhelmed with the challenges associated with caring for patients, the toolkit gave them autonomy to appropriate and use it to suit their conditions. Thus, instead of imposing itself, the toolkit called for cooperation and coordination between the relatives and clinicians in spite of their different backgrounds. This gave form and structure to the use of the toolkit to achieve the intended objectives which can be traced to BO as they are renowned for creating a common understanding without losing the diversity of their social worlds.^32^ This rendered the toolkit as a flexible self-help tool. The relatives could read each chapter and explore worksheets as well as distill different meanings which were sufficiently structured to provide the necessary support.

Thirdly, the dynamic nature of the toolkit as depicted in the study made it easier for both the relatives and therapists to build and sustain their relationship. In mental healthcare, it is the patient–clinician relationship which is prioritised rather than the one involving relatives. Without the toolkit, it was difficult for the therapists to interact with the relatives and derive benefits from it as the information flow became chaotic. The toolkit allowed the interaction to be focused enough to avoid losing direction, but at the same time flexible to respond to the pertinent needs of the relatives. Thus, it was possible to navigate from the broad to the more customised applications that transverse both geographical and knowledge divides. This resonated with the concept of BO^26^ that it is dynamic in maintaining permeable boundaries, which allow diverse forms of knowledge to flow back and forth across social groups. This attribute made it possible for the relatives coming from different professional backgrounds to derive value from REACT-NOR as it served as a general-purpose kit which was malleable to cater for different needs of the users. The dynamism of BOs renders it fairly unstructured when used jointly by different social groups and highly structured when used within a specific situation.^27^ The toolkit did not offer a one size fits all solutions, but was flexible enough to allow the relatives of the patients to appropriate it for their own use.

Fourth, the toolkit brought convenience to the interaction between the relatives and clinicians. By providing a platform for digital meetings, the kit enabled the relatives to get the support they needed in the comfort of their homes and offices. Time and travelling expenses were saved. This function of bringing convenience is not articulated directly in the literature on BO but it is related to the concept of affordability^41^ in terms of the opportunities for virtual communication that the toolkit brings to the caregivers and supporters. The actionable possibilities or affordances^43^ were inherent in the properties of REACT-NOR toolkit, particularly the video function enhanced the experiences of the caregivers and supporters by allowing them to share screens and work inside the toolkit together.

Based on the previously described themes and the implementation process of REACT-NOR, we find that the toolkit embodies the following characteristics that are inherent in BOs:

1. Shared structure: The toolkit provides a platform to link and interact in real-time. It serves as a spanning object whereby the unmet needs of the caregivers are addressed and met by supporters who are able to engage in a dialogue across knowledge domains. The shared structure makes out a virtual space inhabited by both parties.
2. Interpretive flexibility: The toolkit gives caregivers’ autonomy to appropriate and use it to suit their conditions, which will vary widely between families. However, it is resilient enough to convey knowledge resulting in a mutual understanding about psychosis across disciplines and families, and thus calls for alignment and work cooperation.
3. Dynamic nature: The toolkit allows the interaction to be focused enough to avoid losing direction, but at the same time flexible enough to respond to the pertinent needs of the caregivers. It provides psychoeducation spanning from general information to tailored problem-solving.
4. Standardisation: Content, education of supporters, secure video channels and basic therapeutic skills are streamlined to ensure that REACT-NORV does not lose meaning or is altered, and to avoid inconsistencies and potentially conflicting interests. This is accomplished by a rigorous implementation package including supervision of supporters after start up.

However, it can be argued that although the notion of BO is associated with the formation of communities of practice, it is too early to state the extent of new practice formation in this case. However, based on our (Early Intervention in Psychosis Regional Advisory Unit) extensive experience with organising the teaching of regular family educational work to clinicians, we experience that REACT-NOR has been easier to motivate clinicians to attend to. Partly because it is less time-consuming and they find it easy to use compared to the extensive manual in regular family therapy. In addition, the national family network has REACT-NORV on the agenda twice each year when they meet. There are also local groups with supporters who share their experiences.

Limitation: As healthcare is a highly complex and dynamic field with numerous stakeholders, the use of the concept of BO stands the risk of oversimplifying or overlooking the intricate context-specific nuances, diverse perspectives, and unique requirements that characterise different healthcare settings. In addition, the emphasis on standardisation and reductionism by focusing on shared concepts and common ground in the use of BO may undermine the need for customisation and tailored approaches to healthcare delivery, where context-specific factors and personalised care play a crucial role. The current study design was not able to tease out these components. Both relatives and supporters were very pleased with the program. The lack of negative comments may indicate that there are voices we were unable to incorporate in this study. We might also lack the voices of people who are negative to take part in digital services. Digitalisation of healthcare based on the drive to cut costs, can force people to receive services they do not want. Teasing out how BO works in wider settings to be able to accommodate for this, will be important to inform service design strategies.

## Conclusion

The study has revealed the extent to which REACT- NOR tool serves as a BO that connects caregivers and supporters. The tool embodies attributes that define a BO in that it allows the transfer of knowledge and facilitates cross-disciplinary work and collaboration. By virtue of being able to connect, engage and share knowledge, the toolkit in combination with a video provided the interface for the two groups to interact virtually in real-time. This made it possible for both the caregivers and supporters to transverse the geographical and knowledge divide. The capabilities of the toolkit and video solution to inhabit intersecting social worlds resonates with the concept of BO. It was flexible enough to allow the relatives of the patients to appropriate it, dynamic to maintain permeable boundaries for interpretation and communication, structured to enable the participating groups to share meaning and yet standardised to facilitate work across different contexts. With eHealth becoming a prominent source for service delivery, BO theory may provide us with deeper insights into understanding the deployment of digital tools in different contexts. In particular, an understanding of how BO work is crucial with regard to remote care as the transfer of knowledge involves multiple processes such as sharing of new and existing knowledge, translation to make it accessible to others and transformation to render it usable across different boundaries. What emerges from this study is that REACT-NOR provided an arena for sharing knowledge that met the different needs of the caregivers and supporters. The shared space and structure of the toolkit were aligned with the interests of the caregivers and supporters, which made the interaction purposeful. As this study has focused on the use of the REACT-NOR toolkit in the collection and management of knowledge which is subsequently distributed, the notion of BO has been approached as an artefact which serves as a coordination mechanism. It is recommended that further studies should focus on the use of the concept of BO as an analytical lens to disambiguate it from a material construct.

## Supplemental Material

sj-docx-1-dhj-10.1177_20552076231196970 - Supplemental material for Crossing boundaries in the delivery of healthcare – a qualitative study of an eHealth intervention in relation to boundary object theory Click here for additional data file. Supplemental material, sj-docx-1-dhj-10.1177_20552076231196970 for Crossing boundaries in the delivery of healthcare – a qualitative study of an eHealth intervention in relation to boundary object theory by Trust Saidi, Erlend Mork, Sofie Aminoff, Fiona Lobban and Kristin Lie Romm in DIGITAL HEALTH
