# Supplementary material for: Crossing boundaries in the delivery of healthcare – a qualitative study of an eHealth intervention in relation to boundary object theory
Source: Digit Health. 2023 Aug 30;9:20552076231196970. doi: 10.1177/20552076231196970 (PMC10474790; doi:10.1177/20552076231196970)
Supplement: sj-docx-1-dhj-10.1177_20552076231196970 - Supplemental material for Crossing boundaries in the delivery of healthcare – a qualitative study of an eHealth intervention in relation to boundary object theory [file sj-docx-1-dhj-10.1177_20552076231196970.docx]

**INTERVIEW GUIDE**

**A boundary object for supporting relatives of patients with psychosis - A case study of a self-management toolkit in Norway**

**Introduction**

The research aims at exploring how the use of a web-based support programme called Relatives Education and Coping Toolkit (REACT) is used to support family and friends living with psychosis patients. The study uses the toolkit as an object for empowering the relatives of patients with psychosis to cope with stressful situations which they find themselves in. We investigate the use of the technology through the lens of a “boundary object” focusing on how it serves as an intermediary between therapists and relatives of patients. These two groups occupy different knowledge domains that exist between culturally and geographically defined boundaries i.e. home and hospitals. To achieve this, we open for inquiry the “black box” of the toolkit and explore both its contents and structure, paying particular attention to how it connects the two stakeholders. Data will be collected by means of semi-structured interviews that target the therapists and relatives of the patients. Here are the questions that will guide the interviews.

**Questions for Supporters**

- How does REACT-NOR facilitates collaboration with the caregivers of the patients with psychosis?
- How is REACT-NOR designed to empower the relatives of patients to handle stressful situations which they find themselves in?
- How did you experience meeting relatives on the screen instead of face-to-face interaction? Was it difficult or easy, please explain.
- What are your feelings on the extent to which REACT-NOR helps the relatives of the patients in coping with stressful situations?
- Do you think the technology makes it easier or more difficult for the relatives to convey stressful thoughts? Please elaborate.
- What have you learnt from the development and subsequent use of REACT-NOR in assisting the relatives of patients to manage their situation?
- Which part of REACT-NOR programme do you find a) useful b) less useful?
- Nowadays people tend to be overwhelmed with health information from the internet. How different is the information provided by REACT -NOR from the one which can be searched from the internet?
- REACT-NOR as an online tool support measures such as social/physical distancing taken to prevent the spread of COVID 19. What are the benefits of using REACT-NOR toolbox to the caregivers particularly during the time of a pandemic?
- If the use of REACT-NOR was to be withdrawn for some time, what would you miss from it?

**Questions for caregivers**

- How does the use of REACT-NOR equip you to cope with situations of living with patients suffering from psychosis?
- Can you explain with the aid of examples how REACT-NOR has helped you to connect with the therapists?
- To what extend has your situation changed since you started using REACT-NOR?
- Were you familiar with using digital tools such as zoom and teams before you started using REACT-NOR?

If YES, how did your previous experience with digital technologies assist you in using REACT?

If NO, are you less afraid to connect to people digitally than before using REACT-NOR?

- Was there anything that made the use of the webpage easier or difficult for you?
- What can you add or remove from REACT-NO to improve its effectiveness in supporting you?
- Suppose you did not have the opportunity to use REACT-NOR, what do you think your situation will be like?
